# Transient transgenesis of the tapeworm *Taenia crassiceps*

**DOI:** 10.1186/s40064-015-1278-y

**Published:** 2015-09-15

**Authors:** Bárbara Moguel, Norma Moreno-Mendoza, Raúl J. Bobes, Julio C. Carrero, Jesús Chimal-Monroy, Martha E. Díaz-Hernández, Luis Herrera-Estrella, Juan P. Laclette

**Affiliations:** Institute for Biomedical Research, Universidad Nacional Autónoma de México, Av. Universidad 3000, Coyoacán, C.P. 04510 México DF, México; National Laboratory of Genomics for Biodiversity-cinvestav, Km 9.6 Libramiento Norte Carretera Irapuato-León, C.P. 36821 Irapuato, Gto México

**Keywords:** *Taenia crassiceps*, *Taenia solium*, Cysticerci, Transfection, Cytomegalovirus promoter, Green fluorescent protein

## Abstract

Human and porcine cysticercosis is caused by the larval stage of the flatworm *Taenia solium* (Cestoda). Infestation of the human brain, also known as neurocysticercosis, is the most common parasite disease of the central nervous system worldwide. Significant advances in the understanding of the disease have been achieved using the *Taenia crassiceps* murine model. We describe here a successful transfection protocol of *T. crassiceps* cysticerci as the first step to approach a number of currently inaccessible biological questions on cysticercosis. *T. crassiceps* cysticerci (ORF strain) were microinjected with the plasmid pcDNA3.1/NT-GFP-TOPO, encoding the green fluorescent protein (GFP) driven by a cytomegalovirus promoter (CMV). Twelve hours after the microinjection, GFP fluorescence gradually developed in patches associated to bud structures in the bladder wall of cysts. Fluorescence reached a peak at 24–48 h and lasted up to 72 h after the microinjection. Immunohistochemical studies on tissue sections of transfected cysts using an anti-GFP antibody, demonstrated co-localization of the antibody and the GFP fluorescence in the tegumentary cytoplasm and subtegumentary cytons. To validate at the mRNA level the expression of GFP, we carried out RT-PCR using two pairs of nested primers. Results showed expression of GFP-mRNA at 24 h post-transfection. Moreover, western blot assays of crude extracts of transfected cysts, carried out using the anti-GFP specific antibody, showed the expected protein band of 27 kDa, demonstrating that the GFP expression started at 24 after plasmid microinjection and was maintained up to 72 h. These findings will facilitate the development of functional genomics approaches applied to this model of cysticercosis.

**Electronic supplementary material:**

The online version of this article (doi:10.1186/s40064-015-1278-y) contains supplementary material, which is available to authorized users.

## Background

Human and porcine cysticercosis is caused by the larval stage of the tapeworm *Taenia solium* (Cestoda). Infestation of the human brain, known as neurocysticercosis, is the most common parasite disease of the central nervous system worldwide (Garcia et al. 2007). During the last two decades considerable advances in the understanding of cysticercosis have been achieved using a murine model for cysticercosis, which is based on *T. crassiceps*, a close relative of *T. solium* (Sciutto et al. 2011). This cestode naturally infects arctic and red foxes, wolves and dogs as definitive hosts and small rodents including mice as intermediate hosts (Willms and Zurabian 2010). However, several human infections by *T. crassiceps* have been reported, involving both immunocompromised and immunocompetent patients. *T. crassiceps* has been reported in France and other European countries, especially Germany and Norway, as well as in North America and Eastern and Northern Asia (Francois et al. 1998; Stien et al. 2010). The metacestodes have been found in different tissues including subcutis, muscle and remarkably the cerebellum of intermediate hosts (Francois et al. 1998; Ntoukas et al. 2013). *T crassiceps* has been used for several decades as a murine model of cysticercosis, due to the facility of maintenance under laboratory conditions, through intraperitoneal passage from infected into naïve mice (Freeman 1962). Unlike *T. solium*, the metacestodes of this species have the ability to multiply through budding. Two strains of *T. crassiceps* have been maintained under laboratory conditions: WFU and ORF; of which the latter grows faster but does no longer form scoleces (Dorais and Esch 1969; Everhart et al. 2004).

Several reports have described methods for transient and stable transfections in trematode parasites (Boyle and Yoshino 2003; Kalinna and Brindley 2007; Pearce and Freitas 2008; Yang et al. 2010). In cestodes, transient transfection and RNAi silencing have been described for *Echinococcus multilocularis*, *Hymenolepis microstoma and Moniezia expansa* (Brehm and Spiliotis 2008; Pierson et al. 2010; Pouchkina-Stantcheva et al. 2013; Spiliotis et al. 2008, 2010). The availability of accessible, effective and reproducible transfection protocols applied to several Taeniid species would open the possibility of genetic manipulation for these cestodes, improving our ability to address key biological questions on these parasites. The goal of this work was to develop a reproducible method for transient transfection of *T. crassiceps* cysts. An advantage to develop gene transfer protocols for platyhelminths is the availability of the whole genome sequence for several species, including the free-living planarian *Schmidtea mediterranea* (Robb et al. 2008), the trematode parasites *Schistosoma mansoni*, *S. japonicum*, *S. haematobium*, (Berriman et al. 2009; Young et al. 2012; Zhou et al. 2009), and the cestodes *Spirometra erinaceieuropa* (Bennett et al. 2014), *Echinococcus granulosus* (Tsai et al. 2013; Zheng et al. 2013)*, E. multilocularis*, *T. solium* and *Hymenolepis microstoma* (Tsai et al. 2013). These studies have provided a reasonable understanding on the genome and gene structure in these organisms. However, functional genomics tools are now needed to assign the physiological roles of protein-coding genes, and hence identifying novel drug and/or vaccine targets.

Here we report a successful transient transfection protocol for *T. crassiceps* cysts (ORF strain) microinjecting the plasmid pcDNA3.1/NT-GFP-TOPO (Invitrogen), and detecting the expression of GFP through fluorescence microscope observation. Expression of GFP was confirmed at the RNA level by RT-PCR, and at the protein level by immunohistochemistry on tissue sections and western blot on protein extracts, using anti-GFP antibodies.

## Methods

### Transfection of *T. crassiceps* cysticerci by GFP-TOPO plasmid microinjection

All experiments were carried out using cysticerci of *T. crassiceps ORF* strain, characterized by the absence of scolex, which were maintained through intraperitoneal passage from mouse to mouse using 8 weeks old Balb/cAnN females (Sciutto et al. 2011). Cysts were collected from the peritoneal cavity of 3 months infected mice after humanitarian sacrifice, and maintained in vitro at 37 °C under 5 % CO_2_, for 24 h in RPMI medium supplemented with 10 % Fetal Bovine Serum and 1 µg/mL penicillin/streptomycin (Vazquez-Talavera et al. 2001). All procedures involving mice were carried out according to the guidelines of the Biomedical Research Institute, UNAM, and were approved by the Committee for the Care and Use of Experimental Animals (ID 117).

For transfection, cysticerci of 4–5 mm diameter were selected. Microinjection needles were made from commercially available glass capillaries (Glass thinW, World Precision Instruments, Inc. TW100F-6), using a vertical pull type PC-10 (Narishige, Japan). Then, microinjection needles were loaded with 3 µg/µL of plasmid DNA in water with traces of Indian ink. The plasmid employed for the transfection was the commercially available vector pcDNA3.1/NT-GFP-TOPO (Invitrogen), which includes a GFP reporter gene and a neomycin resistance cassette under the control of a CMV promoter region. This plasmid is here referred to as GFP-TOPO. The living cysts were immobilized in 5 mm holes carved in LB agar Petri dishes, adding 50µL of the culture medium to prevent desiccation. Each cysticercus was microinjected with 1 µL (~3 µg of plasmid DNA) into the vesicular fluid, using an Eppendorf Cell Tram Microinjector. Some cysts were microinjected with 3 µg of the pCMV-VSV-G plasmid (Addgene) in 1 µL of sterile water (lacking the coding sequence for GFP), or with the water alone, to be used as negative controls. Afterwards, the larvae were transferred back to the culture medium and maintained in vitro as described above. All cysts survived in culture for at least 1 week after microinjection.

Cysts were checked for fluorescence every 6 h after transfection. To reduce motility during observation and image capture using an Olympus DSU confocal microscope, coupled to a fluorescent lamp with FITC filters (excitation/emission 450–490/515 nm), complete microinjected cysts were placed in excavated cover glasses and maintained on ice for 30 min. Exposure time was identical for the photography of the GFP-TOPO microinjected and of the negative control. To determine the difference between endogenous autofluorescence and GFP fluorescence, quantitative measurements on five plasmid microinjected and five water microinjected cysticerci were carried out every hour during 1 day, using a Modulus II Microplate Multimode Reader (Turner Biosystems) with excitation at 478 nm and emission at 507 nm. This experiment was repeated three times and Student’s *t* test were performed to determine statistical significance.

For optimal microphotography, the microinjected cysts were fixed with 4 % paraformaldehyde in phosphate buffered saline (PBS), pH 7.2, at RT during 20 min and rehydrated overnight in 4 % sucrose in PBS. Afterwards, they were mounted on slides with diazabicyclo (2.2.2.) octane (DABCO 2.5 %) using excavated cover glasses. Intact fluorescent cysts were photographed under a confocal Zeiss microscope (LSM5Pascal; Carl Zeiss).

### Tissue immunohistochemical studies

Cysticerci microinjected with GFP-TOPO, pCMV-VSV-G, or water were processed for immunohistochemical assays after 24 h. Tissue sections from testis of ubiquitously GFP-expressing (B5-GFP) transgenic mice, were included as positive controls (Hadjantonakis et al. 1998). All tissue samples were fixed as described above. The cysts were placed in OCT (optimal cutting temperature) medium (Tissue-Tek; Sakura Finetek) and frozen in hexane (J.T. Baker) on dry ice. After cutting, 20 μm thick sections were permeated with 0.1 % Triton X-100 (Sigma-Aldrich) in PBS for 10 min and then blocked with bovine serum albumin (Gibco) at 1 % in PBS for 2 h. Sections were then incubated overnight with a 1:250 dilution of the polyclonal *α*-GFP rabbit IgG antibody (*Life technology*). After four washes with PBS, sections were incubated at room temperature for 1 h with a 1:200 dilution of goat α-rabbit IgG CY3-conjugated (*Life tech*) antibody. Finally, all sections were mounted in permanent fluorescence mounting medium (Dako Cytomation) and stored at 4 °C for subsequent inspection and photography under confocal microscopy. Serial sections of each tissue sample were incubated in parallel without primary antibody to check for unspecific binding of the secondary antibody. All sections were examined and photographed using a confocal microscope (LSM5Pascal; Carl Zeiss), equipped with argon–krypton and helium–neon lasers, using FITC and Cy3 filters and Nomarski interference contrast.

### RT-PCR analysis

GFP transcription was confirmed by RT-PCR in a two steps reaction using total RNA isolated with Trizol/Chloroform from all microinjected cysts 24 h after transfection. Reverse transcriptase cDNA synthesis on total RNA using oligo-dT primers, was carried out using SuperScript^™^ III First-Strand (Invitrogen), followed by a nested PCR with Hot StarTaq DNA polymerase (Quiagen), using two sets of GFP primers: forward 5′ AAGGAGAAGAACTTTTCACTGG and reverse; 5′CCGTACCTACTCGAGATGTTT for the initial amplification of a 707 bp fragment, and a second pair: 5′ AAGGAGAAGAACTTTTCACTGG and 5′GTTTTAAGCGGTGTTGTAAC, for the amplification of a 228 bp fragment. Total RNA isolated from Hek 293 cells (Graham et al. 1977), transfected by lipofection with GFP-TOPO, was also included as positive control. Electrophoresis of PCR products was carried out in 1 % agarose gels in TAE (Tris–Acetate-EDTA) with 0.25 mg/mL Ethidium Bromide (Bio-Rad Laboratories). Gels were visualized and photographed with a Bio-Imaging System (MiniBis pro, DNS). In order to rule out the possibility that the GFP-TOPO plasmid used for microinjection could remain as template during the RT-PCR described above, separate nested PCR amplifications were carried out (excluding the reverse transcriptase reaction), using the same two sets of primers and the same total RNA isolated from the plasmid microinjected cysts.

### Western blot analysis

**Fig. 1:**
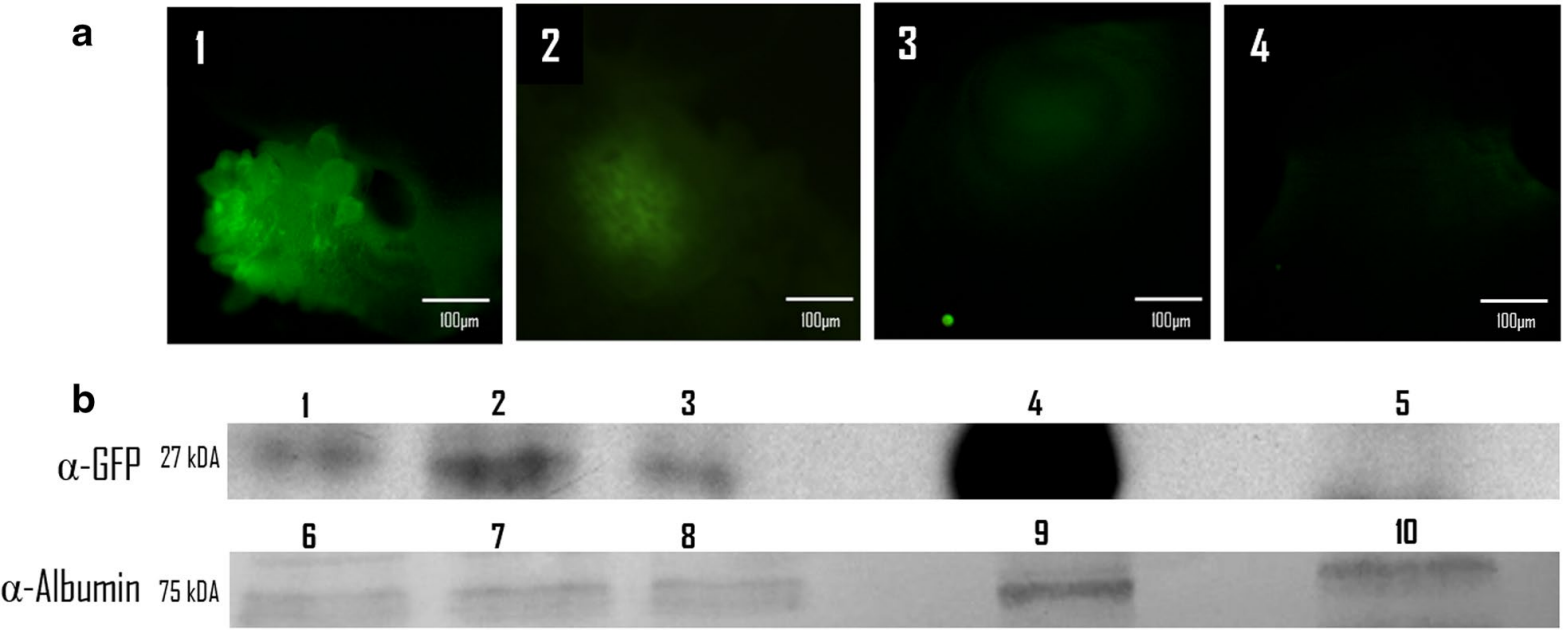
Time course of the GFP fluorescence after microinjection of intact *Taenia crassiceps* cysticerci. **a** GFP-TOPO plasmid (*1*–*3*) and a GFP-negative plasmid: pCMV-VSV-G (*4*) microinjected cysts, after 24 (*1* and *4*); 48 (*2*) and 72 h (*3*). Photographs were taken using an Olympus DSU confocal system with a FITC (450–490 nm) filter under the same conditions for all cases. **b** Western blots using crude extracts of GFP-TOPO (*1*–*3*) and pCMV-VSV-G (*5*) microinjected cysts; 24 (*1* and *5*); 48 (*2*) and 72 (*3*) h post microinjection. A crude extract of HEK 293 cells transfected with GFP-TOPO (*4*) is also shown as a positive control. 50 µg of each crude extract were loaded on each lane in the gel and all blots were obtained from a single gel run. For detection of GFP, a polyclonal rabbit IgG α-GFP antibody and a goat α-rabbit IgG antibody conjugated with horseradish peroxidase were used at 1:1000 and 1:10,000 dilutions, respectively. Loading controls (*6*–*10*) used a sheep anti-mouse albumin polyclonal antibody followed by a horseradish peroxidase conjugated anti-sheep IgG secondary antibody (Aldridge et al. 2006). Development of peroxidase was carried out using an enhanced chemiluminescence kit

Western blotting was performed to evaluate the presence of GFP in the transfected fluorescent cysts. Crude protein extracts were obtained 24, 48 and 72 h after microinjection of cysts with GFP-TOPO, pCMV-VSV-G, or water, as well as from transfected Hek 293 cells. Homogenization was carried out using 2 % Laemli buffer: 50 mM Tris–HCl (pH 6.8), 100 mM dithiothreitol, 2 % SDS, 0.1 % bromophenol blue and 10 % glycerol in water. Proteins were resolved by SDS-PAGE using 15 % polyacrylamide gels and blotted onto nitrocellulose membranes. In each experiment, the protein extracts obtained at different times after transfection were loaded in a single gel for electroblotting. 50 µg total protein of each crude extract were loaded on each lane in the gel and all blots were obtained from a single gel run. Membranes were blocked by overnight incubation at 4 °C with 10 % dry skimmed milk in PBS buffer, and then incubated with Peroxidase Blocking Reagent (Biocare Medical) for 15 min at room temperature. The membranes were incubated with the first and the second antibodies for 2 h each, at 37 °C. Polyclonal α-GFP antibody (*Life technology*) and horseradish peroxidase conjugated α-rabbit antibody (INVITROGEN) were used at 1:1000 and 1:10,000 dilutions, respectively. Loading controls for western blots of *T. crassiceps* crude extracts were carried out using host albumin for reference (Aldridge et al. 2006); a sheep anti-mouse albumin polyclonal IgG antibody (Abcam) was used followed by a horseradish peroxidase conjugated anti-sheep IgG (Abcam) secondary antibody. In all cases, development of peroxidase reaction was done using a enhanced chemiluminescence kit (Amersham, NJ, USA). Membrane exposure for the protein extracts shown in Fig. 1 was 10 s.

## Results

### GFP Fluorescence was evident in GFP-TOPO microinjected cysticerci

Around 40 % of the *T. crassiceps* cysticerci microinjected in the vesicular fluid with GFP-TOPO plasmid showed green fluorescence in patches on the bladder wall (Fig. 1a). Frequently, the fluorescent patches appeared in the buds of the plasmid microinjected cysts, however, in fewer cases the fluorescent patches appeared in other parts of the vesicle. Another interesting observation was that not all buds resulted fluorescent after microinjection of a cyst with GFP-TOPO. In contrast, low autofluorescence signal was observed in water microinjected cysts (Fig. 1a). Fluorescence was evaluated during 72 h after transfection. Maximal GFP fluorescence was observed at 24 h post microinjection and faded out between 48 and 72 h (Fig. 1a). The difference between autofluorescence and the GFP signal was quantitatively evaluated every hour during 24 on five GFP-TOPO plasmid and five water microinjected cysts. This experiment was repeated three times. Statistical t Student tests showed highly significant differences (p < 0.05) in fluorescence at 507 nm between both groups of cysts (Additional file 1: Figure S1).

### GFP was immunolocalized in the tissue of GFP-TOPO microinjected cysticerci

**Fig. 2:**
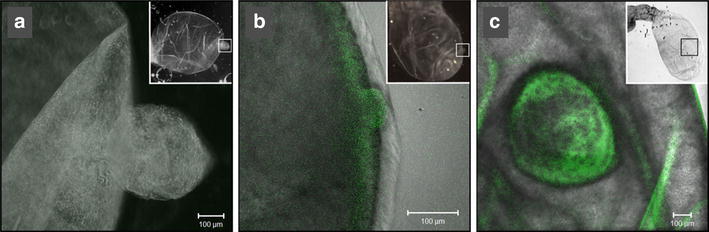
GFP fluorescence in budding areas of the bladder wall after microinjection of intact *T. crassiceps* cysticerci. **a** Water microinjected cyst; **b**, **c** GFP-TOPO plasmid microinjected cyst, lateral and frontal views, respectively. Photographs were obtained in a confocal microscope with a FITC (450–490 nm) filter. *Upper right squares* show images of the corresponding complete cysts under stereo microscopy

A more detailed observation of the plasmid microinjected cysts was carried out under confocal microscopy on the bladder walls in toto, after paraformaldehyde fixation. As described above, the GFP fluorescence was located in discrete patches mainly associated with budding regions of the bladder wall (Fig. 2b, c), whereas cysticerci microinjected with water showed low levels of autofluorescence (Fig. 2a).

**Fig. 3:**
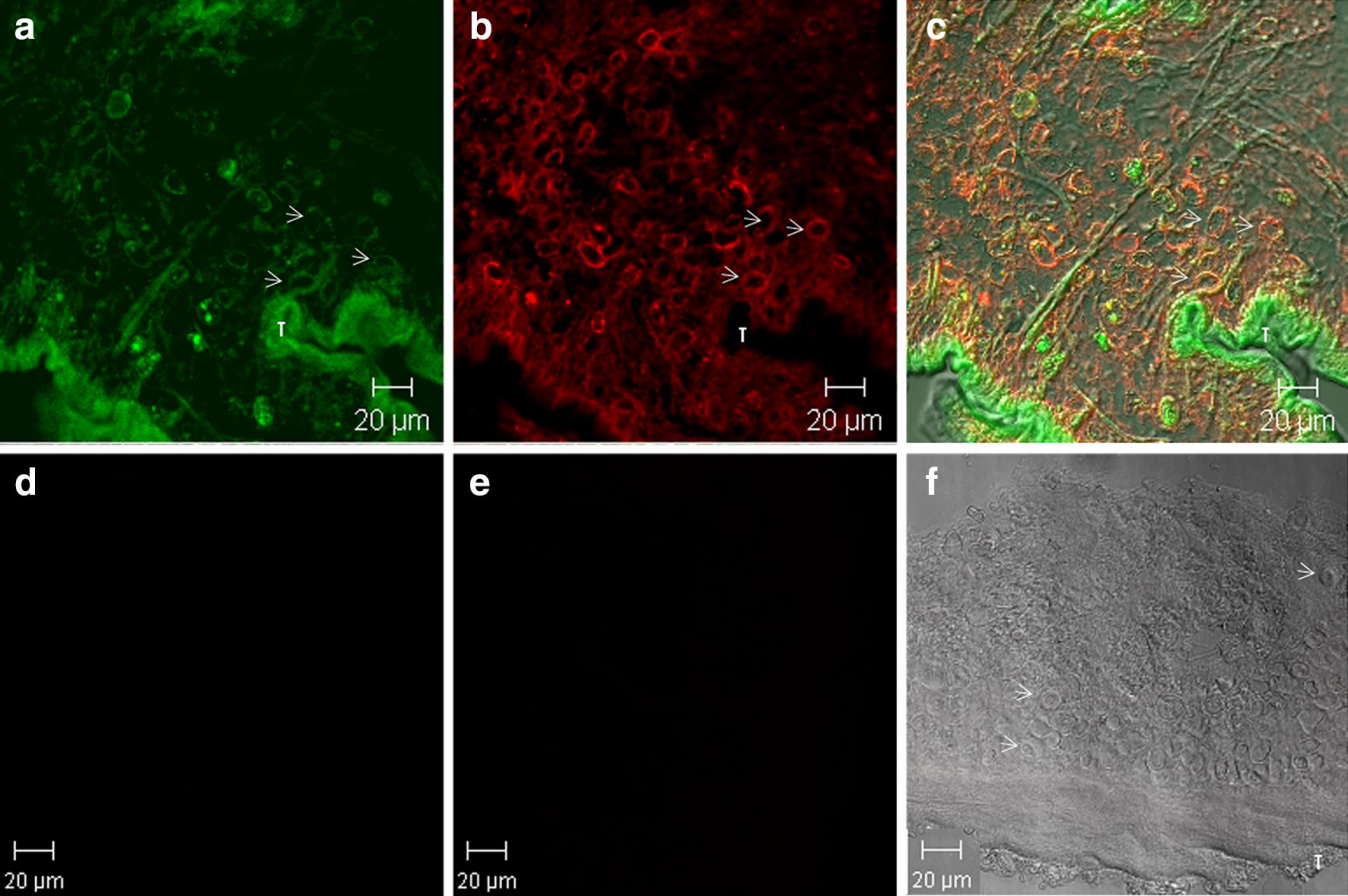
Co-localization of GFP fluorescence and α-GFP immunofluorescence in tissue sections of cysticerci after 24 h of microinjection. **a** GFP fluorescence in a GFP-TOPO plasmid microinjected cyst (FITC filter); **b** Immunohistochemical localization using a 1:250 dilution of a polyclonal *α*-GFP rabbit IgG antibody followed by a 1:200 dilution of a goat α-rabbit IgG CY3-conjugated antibody (CY3 filter); **c** Merging of **a** and **b** images. **d** Water microinjected cysts (FITC filter); **e** Immunohistochemical localization as in **b** in a water microinjected cyst (CY3 filter); **f** Merging of **d** and **e** images. *Arrows* show subtegumentary cytons. *T* tegument. Photographs were obtained under Confocal Laser microscopy

The intracellular distribution and localization of GFP were studied on tissue sections of GFP-TOPO, pCMV-VSV-G, or water microinjected cysts observed in the confocal microscope. The images clearly showed that GFP was only expressed in the tegumentary cytoplasm and sub-tegumentary cytons of the GFP-TOPO microinjected cysts (Fig. 3a). In order to determine the tissue localization of GFP, immunohistochemical assays were performed on serial tissue sections using a polyclonal rabbit IgG α-GFP antibody and a fluorescent goat α-rabbit IgG-CY3 conjugate (Fig. 3b). Results showed overlapping between the red fluorescence of the α-GFP antibody and the green GFP fluorescence described above in the tegumentary cytoplasm and in the sub-tegumentary cytons of the bladder wall (Fig. 3c). The antibody recognition of GFP in the tegument appeared slightly weaker than in the sub-tegumentary cytons, suggesting either than the level of GFP was lower in the tegumentary cytoplasm or that the permeation carried out on the tissue sections using Triton X-100 was not equally efficient in the tegument region that is essentially made up of a large amount of micro-vesicles and mitochondria. In the case of pCMV-VSV-G or water microinjected cysts, no fluorescence was observed either by itself or using the polyclonal α-GFP antibody and the second antibody (Fig. 3d–f). Sections from GFP-TOPO microinjected cysticerci incubated with the second antibody alone resulted entirely negative (Additional file 2: Figure S2). Moreover, similar immunohistochemical assays using an unrelated first antibody (i.e. a polyclonal α-DDX4/VASA), directed to a stem cell marker, resulted in an entirely different pattern of fluorescence in the cyst’s tissue when the same secondary antibody was applied (Additional file 3: Figure S3), excluding the possibility of unspecific anti-GFP fluorescence. Tissue sections from testis of ubiquitously GFP-expressing (B5-GFP) transgenic mice, were also used as positive controls for the fluorescence follow up (not shown).

### Expression of GFP in GFP-TOPO microinjected cysticerci

**Fig. 4:**
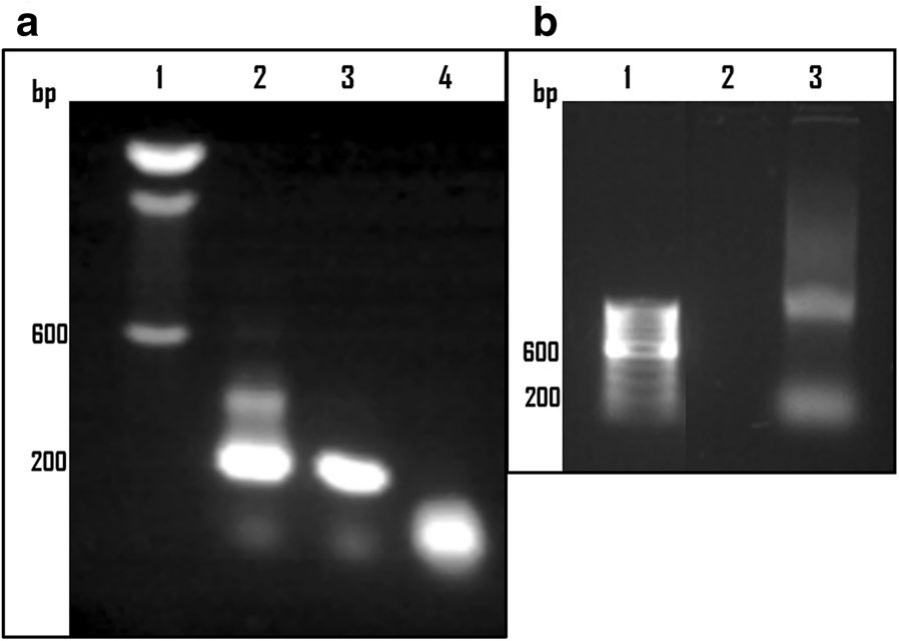
GFP transcription in *T. crassiceps* microinjected cysticerci. **a** RT-PCR performed on total RNA from GFP-TOPO or water microinjected cysts and lipofectamine transfected HEK 293 cells; RNA was isolated 24 h post microinjection/transfection, then, a nested PCR amplification was made using two sets of GFP primers. *Lanes*: *1* DNA size markers; *2* HEK 293 cells transfected with GFP-TOPO plasmid; *3* and *4* *T. crassiceps* cysts microinjected with GFP-TOPO plasmid or water, respectively. **b** Plasmid contamination control for the total RNAs used in **a**. *Lanes*: *1* DNA size markers; *2* and *3* Nested PCR amplifications (excluding the reverse transcriptase reaction) using the same sets of GFP primers on total RNA from *T. crassiceps* cysts microinjected with GFP-TOPO plasmid, and from HEK 293 GFP-TOPO transfected cells, respectively

Transcription of GFP encoded in the GFP-TOPO plasmid in the microinjected cysts was determined by nested RT-PCR. Results showed a single band of 228 bp in the plasmid transfected cysts in agreement with the expected fragment size in our nested RT-PCR (Fig. 4a). Similar RT-PCR on HEK 293 cells transfected with the same plasmid, used as positive controls, showed an identical band of 228 bp (Fig. 4a). The bands from the GFP-TOPO microinjected and from the HEK 293 transfected cells were sequenced and 100 % identity was found between both PCR products and the reported sequence for GFP (not shown). In contrast, no PCR band was detected in the water microinjected cysts (Fig. 4a). The possibility that the plasmid used for microinjection could remain as template during the RT-PCR, was explored in separate nested PCR amplifications, using the same two sets of primers and the same total RNA isolated from the plasmid microinjected cysts. No amplified band was observed for the GFP-TOPO microinjected cysts (Fig. 4b). In the commercial plasmid that we used, expression of GFP is supposedly driven by the CMV promoter. However, the participation of other promoter structures in the plasmid cannot be ruled out.

GFP expression was also evaluated by western blot on crude extracts from GFP-TOPO, pCMV-VSV-G, or water microinjected cysts using anti-GFP antibodies. The expected 27 kDa band corresponding to GFP was detected in the crude extracts of the GFP-TOPO plasmid microinjected cysts. In contrast, no GFP expression was detected in the pCMV-VSV-G or water microinjected larvae (Fig. 1b). Western blotting confirmed that GFP expression was maintained from 24 to 48 h after transfection, followed by a progressive and gradual decrease up to 72 h (Fig. 1b).

## Discussion

Developing techniques for genetic manipulation has become a relevant issue in flatworm biology (Beckmann and Grevelding 2012; Hoffmann et al. 2014; Kalinna and Brindley 2007; Rinaldi et al. 2012a, b). During the last decades, a number of attempts have been made for the identification and characterization of regions that control the expression of genes in these worms. Besides, the availability of the genome sequence of the free living *Schmidtea mediterranea* (Robb et al. 2008), as well as of the trematodes *Schistosoma mansoni* (Berriman et al. 2009), *S. japonicum* (Zhou et al. 2009), *S. haematobium* (Young et al. 2012), and the cestodes *Spirometra erinaceieuropa* (Bennett et al. 2014), *E. granulosus* (Tsai et al. 2013; Zheng et al. 2013)*, Echinococcus multilocularis*, *T. solium* and *Hymenolepis microstoma* (Tsai et al. 2013), will foster the definition of gene lists and the identification of orthologous genes of each species and group, as well as their functional promoters.

The most intensely transfected flatworms are the schistosomes, where several methodologies have already been implemented for genetic manipulations, such as gene silencing through RNAi or transient and stable transfection of heterologous genes, making possible new approaches to old unsolved questions on these parasites (Hoffmann et al. 2014; Kalinna and Brindley 2007; Rinaldi et al. 2012a; b). Several reviews are available on this topic (Kalinna and Brindley 2007; Mann et al. 2011; Suttiprapa et al. 2012). In the case of other trematodes of high global prevalence such as *Clonorchis sinesis*, *Opisthorchis viverinni*, *Paragonimus spp*, *Fasciolopsis busia* and *Fasciola hepatica*, reports on transfection are scarce (Hotez et al. 2008; McVeigh et al. 2014; Rinaldi et al. 2008; Sripa et al. 2011; Wang et al. 2014). For example, in the case of *Paragonimus westermani*, two retro-transposons of the LTR family (Long Terminal Retrotansposons) have been isolated and characterized, which allowed the selection of genes with ability of integration to the genome (Bae and Kong 2003). In cestodes, transient transfection was carried out in primary cells of *E. multilocularis* using plasmid pEMTF-3 containing a fusion of the cyano-fluorescent-protein (CFP) and the V5 antibody epitope reading frames, under expression control of the *E. multilocularis* elp promoter (Brehm and Spiliotis 2008; Spiliotis et al. 2010). Other reports on cestodes have shown a successful gene suppression using RNAi in *H. microstoma* and *M. expansa* (Pierson et al. 2010; Pouchkina-Stantcheva et al. 2013).

Taeniid metacestodes are syncytial organisms. We used cysticerci of *T. crassiceps* because of the facility for laboratory propagation and maintenance, but especially because of their ability to multiply through budding of the bladder wall, implying that the syncytial cysticerci’s tissue has numerous germinal cytons. A previous study has shown that it is possible to regenerate complete cysticerci from larval cells culture (Toledo et al. 1997). Besides, this species of tapeworm has been used in diverse studies as a model for porcine cysticercosis (Sciutto et al. 2011; Willms and Zurabian 2010).

Several methods were tested for the transfection of intact *T. crassiceps* cysts using the GFP-TOPO plasmid, including electroporation, lipofection and soaking, without success. The successful and reproducible transient transfection reported here, was carried out by microinjecting GFP-TOPO plasmid DNA into the vesicular cavity of 3–5 mm diameter cysts, indicating that the plasmid DNA was spontaneously taken up from the vesicular fluid by the subtegumentary cytons. The ability of Taeniid cysts to uptake intact macromolecules has been known for a long time (Ambrosio et al. 1994; Khalil et al. 1998). However, the precise mechanism remains to be characterized. The cyton types underneath the tegument have been poorly studied in the case of *Taeniasp*. cysts. In this situation, the term “subtegumentary cyton” does not imply a specific cell type but a localization underneath the bladder wall basement membrane.

It is worth mentioning that tapeworm cysts are highly autofluorescent. Autofluorescence of Taeniid cysts in red is due to the presence of large quantities of porphyrin derivatives: protoporphyrin IX, coproporphyrin I or III, uroporphyrin and others (Larralde et al. 1986, 1987). Autofluorescence decay under in vitro culture is apparently due to release of those fluorescent materials into the culture medium, starting at about 12 h and increasing asymptotically up to 6 days (Larralde et al. 1986). Although this was described for *T. solium*, our experiments show that *T. crassiceps* behaves in a similar way.

Autofluorescence of cysts peaks at 582 and 630 nm when excited at 410 nm (Larralde et al. 1986), whereas GFP fluorescence peaks at 507 nm; we used a FITC filter for excitation corresponding to a light path of 460–490 (peak 478 nm) nm. Under this condition, a small overlapping between the GFP fluorescence and the cyst’s autofluorescence takes place, however, this overlapping is not enough to outshine the green GFP fluorescence, as evaluated in our quantitative measurements on three repeats of five plasmid microinjected and five water microinjected cysticerci (Additional file 1: Figure S1).

Our results demonstrated GFP expression in bladder wall regions of active proliferation of cytons during formation of buds. Interestingly, localization of GFP fluorescence was also associated to the syncytial tegument. Because the tegument lacks nuclei and ribosomes, it is possible that GFP biosynthesis occurred in the subtegumentary cytons and was then exported to the tegument, as described for other species of metacestodes (Lumsden et al. 1974). The co-localization of the GFP and the α-GFP antibody fluorescence indicated that the heterologous DNA was transcribed by the GFP-TOPO microinjected cysticerci. Even when this transient transfection was highly reproducible, the level of both GFP mRNA and protein were relatively low. It is possible that a stronger promoter or the inclusion of introns in the GFP construct could enhance the level of GFP expression in the transfected cysts (see below).

To rule out the possibility that a misleading protein expression happened unrelated to the microinjection of the GFP-TOPO, a GFP-negative plasmid was also assayed as control in addition to the water microinjected cysts; experiments were carried out using pCMV-VSV-G plasmid, which has the same CMV promoter but lacks the coding region for GFP. In no case any fluorescence that could be confused with GFP fluorescence was observed in the cysts (not shown). Moreover, we also carried out western blots and immunohistochemical assays (similar to those described here after microinjection of cysts with GFP-TOPO) in these pCMV-VSV-G microinjected cysts. Again, in no case any GFP expression was detected.

The lack of germinal cell lines for *T. crassiceps* led us to approach transfection using intact larvae. However, our current efforts are also directed to the identification and isolation of germinal cells on one side, and to the development of a procedure for the stable transfection of *T. crassiceps* using the PiggyBac transposon and the *T. solium* actin (Correnti et al. 2007; Morales et al. 2007) and splice leader (SL) (Davis et al. 1999; Kines et al. 2006) promoters with GFP as reporter gene. Other novel possibility that could be explored in these parasites is the use of CRISPR/Cas system (Burt 2003; Oye et al. 2014). Nevertheless, the availability of an easy and reproducible transient transfection makes feasible a number of genetic interventions on these organisms, for the elucidation of signaling pathways or protein–protein interactions, etc. A strong basic science is necessary to develop the biotechnological tools required for the control of this and other neglected parasite diseases.

## Additional files

Additional file 1: Figure S1. Line plot of two variables: Fluorescence intensities detected in GFP-TOPO (squares) and water microinjected (circles) *T. crassiceps* cysticerci using a Modulus II Microplate Multimode Reader (Turner Biosystems) with excitation at 478 nm and emission at 507 nm. This experiment was repeated three times using quantitative measurements on five plasmid microinjected and five water microinjected cysticerci evaluated every hour during 1 day. Two tails Student’s t test were performed to determine statistical significance with p value ≤0.01, shown inside the white square. The analysis was performed in STATISTICA 12 software
 Additional file 2: Figure S2. Technique control for the immunohistochemistry carried out on tissue sections of *Taenia crassiceps* cysts after GFP-TOPO microinjection. (A) Merged image of the immunohistochemical localization of GFP (B) using a 1:250 dilution of a polyclonal *α*-GFP rabbit IgG antibody followed by a 1:200 dilution of a goat α-rabbit IgG CY3-conjugated antibody (CY3 filter) and its corresponding Nomarski Interference Contrast image (C). (D) Merged image of the immunohistochemical localization of GFP (E) using a 1:200 dilution of a goat α-rabbit IgG CY3-conjugated antibody (CY3 filter) and its corresponding Nomarski Interference Contrast image (F)
 Additional file 3: Figure S3. Immunohistochemical assay using a polyclonal α-DDX4/VASA antibody directed to a stem cell marker applied on tissue sections of *Taenia crassiceps* cysts. (A) Merged image of the immunohistochemical localization of a stem cell marker using a 1:250 dilution of a polyclonal α-DDX4/VASA using followed by a 1:200 dilution of a goat α-rabbit IgG CY3-conjugated antibody (CY3 filter) and its corresponding Nomarski Interference image. (B) Merged image of the immunohistochemical localization of a stem cell marker using a 1:200 dilution of a goat α-rabbit IgG CY3-conjugated antibody (CY3 filter) and its corresponding Nomarski Interference image
