# Supplementary material for: Transient transgenesis of the tapeworm Taenia crassiceps
Source: Springerplus. 2015 Sep 15;4:496. doi: 10.1186/s40064-015-1278-y (PMC4571025; doi:10.1186/s40064-015-1278-y)
Supplement: Supplementary file 1 — Additional file 1: Figure S1. Line plot of two variables: Fluorescence intensities detected in GFP-TOPO (squares) and water microinjected (circles) T. crassiceps cysticerci using a Modulus II Microplate Multimode Reader (Turner Biosystems) with excitation at 478 nm and emission at 507 nm. This experiment was repeated three times using quantitative measurements on five plasmid microinjected and five water microinjected cysticerci evaluated every hour during 1 day. Two tails Student’s t test were performed to determine statistical significance with p value ≤0.01, shown inside the white square. The analysis was performed in STATISTICA 12 software [file 40064_2015_1278_MOESM1_ESM.docx]

Additional file 1: Figure S1

Transient transgenesis of the tapeworm *Taenia crassiceps*

SpringerPlus

Bárbara Moguel^1^, Norma Moreno-Mendoza^1^, Raúl J. Bobes^1^, Julio C. Carrero^1^, Jesús Chimal-Monroy^1^, Martha E. Díaz-Hernández^1^, Luis Herrera-Estrella^2^, Juan P. Laclette^1*^

^1^Institute for Biomedical Research, Universidad Nacional Autónoma de México

and ^2^National Laboratory of Genomics for Biodiversity-CINVESTAV Irapuato, México.

Corresponding author:

Juan P. Laclette^*^

Institute for Biomedical Research, Universidad Nacional Autónoma de México

Av. Universidad 3000, C.P. 04510, Coyoacán, Distrito Federal, México

e-mail address [laclette@biomedicas.unam.mx](mailto:laclette@biomedicas.unam.mx)


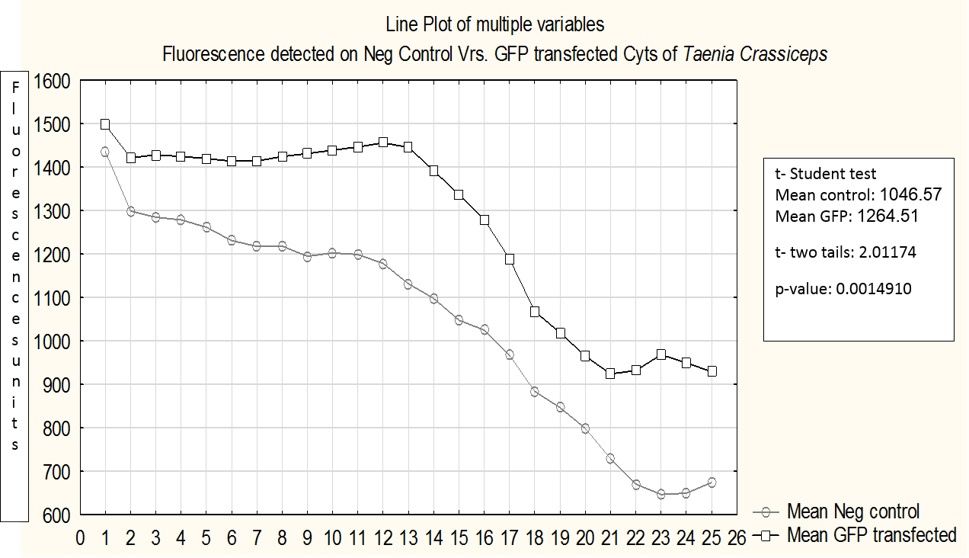


Suppl. Fig 1: Line plot of two variables: Fluorescence intensities detected in GFP-TOPO (squares) and water microinjected (circles) *T. crassiceps* cysticerci using a Modulus II Microplate Multimode Reader (Turner Biosystems) with excitation at 478 nm and emission at 507 nm. This experiment was repeated three times using quantitative measurements on five plasmid microinjected and five water microinjected cysticerci evaluated every hour during one day. Two tails Student's t-test were performed to determine statistical significance with p-value ≤ 0.01, shown inside the white square. The analysis was performed in STATISTICA 12 software.
