# Supplementary material for: Transient transgenesis of the tapeworm Taenia crassiceps
Source: Springerplus. 2015 Sep 15;4:496. doi: 10.1186/s40064-015-1278-y (PMC4571025; doi:10.1186/s40064-015-1278-y)
Supplement: Supplementary file 3 — Additional file 3: Figure S3. Immunohistochemical assay using a polyclonal α-DDX4/VASA antibody directed to a stem cell marker applied on tissue sections of Taenia crassiceps cysts. (A) Merged image of the immunohistochemical localization of a stem cell marker using a 1:250 dilution of a polyclonal α-DDX4/VASA using followed by a 1:200 dilution of a goat α-rabbit IgG CY3-conjugated antibody (CY3 filter) and its corresponding Nomarski Interference image. (B) Merged image of the immunohistochemical localization of a stem cell marker using a 1:200 dilution of a goat α-rabbit IgG CY3-conjugated antibody (CY3 filter) and its corresponding Nomarski Interference image [file 40064_2015_1278_MOESM3_ESM.docx]

Additional File 3: Figure S3.

Transient transgenesis of the tapeworm *Taenia crassiceps*

SpringerPlus

Bárbara Moguel^1^, Norma Moreno-Mendoza^1^, Raúl J. Bobes^1^, Julio C. Carrero^1^, Jesús Chimal-Monroy^1^, Martha E. Díaz-Hernández^1^, Luis Herrera-Estrella^2^, Juan P. Laclette^1*^

^1^Institute for Biomedical Research, Universidad Nacional Autónoma de México

and ^2^National Laboratory of Genomics for Biodiversity-CINVESTAV Irapuato, México.

Corresponding author:

Juan P. Laclette^*^

Institute for Biomedical Research, Universidad Nacional Autónoma de México

Av. Universidad 3000, C.P. 04510, Coyoacán, Distrito Federal, México

e-mail address [laclette@biomedicas.unam.mx](mailto:laclette@biomedicas.unam.mx)

Suppl. Fig 3: Immunohistochemical assay using a polyclonal α-DDX4/VASA antibody directed to a stem cell marker applied on tissue sections of *Taenia crassiceps* cysts. (A) Merged image of the immunohistochemical localization of a stem cell marker using a 1:250 dilution of a polyclonal α-DDX4/VASA using followed by a 1:200 dilution of a goat α-rabbit IgG CY3-conjugated antibody (CY3 filter) and its corresponding Nomarski Interference image. (B) Merged image of the immunohistochemical localization of a stem cell marker using a a 1:200 dilution of a goat α-rabbit IgG CY3-conjugated antibody (CY3 filter) and its corresponding Nomarski Interference image.
